# High-Performance Screen-Printed Thermoelectric Films on Fabrics

**DOI:** 10.1038/s41598-017-07654-2

**Published:** 2017-08-04

**Authors:** Sunmi Shin, Rajan Kumar, Jong Wook Roh, Dong-Su Ko, Hyun-Sik Kim, Sang Il Kim, Lu Yin, Sarah M. Schlossberg, Shuang Cui, Jung-Min You, Soonshin Kwon, Jianlin Zheng, Joseph Wang, Renkun Chen

**Affiliations:** 1 Materials Science and Engineering Program, University of California, San Diego, La Jolla, California, 92093 United States; 2 Department of NanoEngineering, University of California, San Diego, La Jolla, California, 92093 United States; 3 Samsung Advanced Institute of Technology, Samsung Electronics, 130 Samsung-ro, Suwon-si, Gyeonggi-do 63272 Korea; 4 Department of Mechanical and Aerospace Engineering, University of California, San Diego, La Jolla, California, 92093 United States; 5 Department of Materials Science and Engineering, University of Seoul, Seoul, 02504 Korea

## Abstract

Printing techniques could offer a scalable approach to fabricate thermoelectric (TE) devices on flexible substrates for power generation used in wearable devices and personalized thermo-regulation. However, typical printing processes need a large concentration of binder additives, which often render a detrimental effect on electrical transport of the printed TE layers. Here, we report scalable screen-printing of TE layers on flexible fiber glass fabrics, by rationally optimizing the printing inks consisting of TE particles (p-type Bi_0.5_Sb_1.5_Te_3_ or n-type Bi_2_Te_2.7_Se_0.3_), binders, and organic solvents. We identified a suitable binder additive, methyl cellulose, which offers suitable viscosity for printability at a very small concentration (0.45–0.60 wt.%), thus minimizing its negative impact on electrical transport. Following printing, the binders were subsequently burnt off via sintering and hot pressing. We found that the nanoscale defects left behind after the binder burnt off became effective phonon scattering centers, leading to low lattice thermal conductivity in the printed n-type material. With the high electrical conductivity and low thermal conductivity, the screen-printed TE layers showed high room-temperature ZT values of 0.65 and 0.81 for p-type and n-type, respectively.

## Introduction

Thermoelectric (TE) devices have garnered tremendous interests recently due to their promise for energy harvesting and solid-state refrigeration^1–11^. Traditional approaches to fabricate TE typically involve ingot fabrication and dicing, which could be time consuming, energy intensive, and often results in rigid devices. On the other hand, there is increasing interest to fabricate TE devices on flexible substrates and using more scalable approaches, for emerging applications such as wearable TE power generation and personalized thermo-regulation^12–16^. Toward this end, high-throughput and inexpensive printing has emerged as a promising fabrication route and has been widely explored in recent years^17, 18^. Interesting applications have thus been demonstrated with printed TE devices, such as printed TE generators for harvesting human body thermal energy^19^, energy harvesting for wireless sensor network applications^20, 21^, integration with energy storage devices^22^, as wearable TE generator on glass fabric^23^.

The bottleneck of the printed TE devices still lies in the low performance of the printed TE materials. In most processes, the TE particles were often mixed with insulating polymeric binders to make the mixture suitable for printing. A high mass loading of the polymer binders would hinder the electrical conductivity. For instance, Madan and coworkers^20, 21, 24–28^ developed a series of printed thermoelectric-epoxy composite films and showed TE figure of merit (ZT) up to 0.31 and 0.41 for n-type Bi_2_Te_3_^20, 25–27^ and p-type Sb_2_Te_3_ or Bi_0.5_Sb_1.5_Te_3_ based materials^20, 21, 25, 28^, respectively. The mass loading of the polymer binders was up to ~20%. The mixtures were typically cured at 250–350 °C to solidify the films. Lu *et al*.^29^ demonstrated inkjet printing of nanoparticles to fabricate TE films which yielded maximum TE power factor of ∼77 and 183 μW/m-K^2^ at 75 °C for films based on Sb_1.5_Bi_0.5_Te_3_ and Bi_2_Te_2.7_Se_0.3_ nanoparticles, respectively. The relatively low power factor values were attributed to the low electrical conductivity^29^. Navone *et al*.^30, 31^ used screen printing and uniaxial densification at 350 °C to fabricate TE micro-modules on polyimide substrates with p- and n-type pillars with 4 and 6 μW/m-K^2^ in power factor, respectively. Recently, Varghese *et al*.^32^ screen printed n-type Bi_2_Te_2.8_Se_0.2_ nanoplate crystals and achieved a peak ZT of 0.43. We *et al*.^33^ used screen printing to fabricate n-type Bi_2_Te_3_ thick films. They optimized the annealing process and achieved a high ZT of 0.61 with 500 °C annealing for 15 mins. Similarly, Kim *et al*.^34^ achieved a ZT of 0.41 in p-type Sb_2_Te_3_ thick film processed by screen-printing followed by thermal annealing.

Apart from electrically inactive binders, several teams have also attempted to add conductive polymers as binders into active TE materials to improve the electrical conductivity. For example, Kato *et al*.^35^ prepared a mixture of Bi_0.4_Te_3_Sb_1.6_ particles, conductive PEDOT:PSS and PAA (Poly (acrylic acid)), and other organic additives, and then spin-coated the mixture to yield thin films with a ZT of 0.2 at 300 K. Bae *et al*.^36^ enhanced the TE properties of PEDOT:PSS by adding Te nanorods and using chemical treatment. However, the Seebeck coefficient (*S*) of the printed films was usually low due to the low *S* of the conductivity polymers. In addition to Bi-Te based materials, other materials have also been printed. Lee *et al*.^37, 38^ screen-printed ZnSb films and yielded a power factor of 1.06 mW/m-K^2^. They further fabricated a TE module using p-type ZnSb and n-type CoSb_3_ films^38^. Hong *et al*.^39^ used inkjet printing to fabricate TE ZnO and ZnFe_2_O_4_ thin films.

Despite the tremendous efforts on printed TE films, most printed devices are still limited by a relatively low ZT compared to those made from commercial bulk processes: the highest reported ZT of printed TE materials are 0.61 for n-type^33^ and 0.41 for p-type^34^. As mentioned earlier, the primary reason behind the low ZT is the presence of the organic additives, such as binders, needed to make TE slurries with suitable viscosity for printing. If a large amount of these additives were used and not effectively removed, the resultant TE films would be limited by low electrical conductivity, as reported in prior work. One feasible approach to remove the organic binders is thermolysis, namely, decomposition of the organic species via burning. This process typically requires sufficient oxygen at elevated temperatures. However, exposing the TE slurries to oxygen could also oxidize the TE materials and consequently impact the TE properties. Therefore, in order to achieve a high ZT in printed TE films, it is important to: (1) use as little amount of binder as possible while maintaining the suitable viscosity for printability; (2) remove the binder as much as possible without oxidizing the TE components.

In this work, we developed a screen printing process to fabricate high-performance TE layers. We have identified a suitable binder from a class of methyl cellulose or more known for its trade name, Methocel, to make printable TE slurries with a low concentration (0.45–0.60 wt.%), thus minimizing its detrimental effect on electrical transport. Also, it is advantageous that Methocel, which is cellulose ether, can be decomposed in an inert atmosphere, such as Ar, at a temperature that is below the typical hot pressing temperature for Bi-Te alloys (~400–450 °C)^40^. We also achieved high-quality printing of the TE layers on rough and porous fabric substrates by introducing an interface layer before printing the TE layer on the fabrics. Following printing, the binders were subsequently burnt off via sintering and hot pressing. We found reduced thermal conductivity in the n-type printed layer, which can be attributed to phonon scattering by the nanoscale defects formed after burning off the binder. With the high electrical conductivity and low thermal conductivity, the screen-printed TE layers displayed high room-temperature ZT values of 0.65 and 0.81 for p-type Bi_0.5_Sb_1.5_Te_3_ and n-type Bi_2_Te_2.7_Se_0.3_, respectively. These ZT values are among the highest for printed samples, and indicate considerable promise for developing wearable TE devices.

## Results and Discussion

### Screen printing

**Figure 1:**
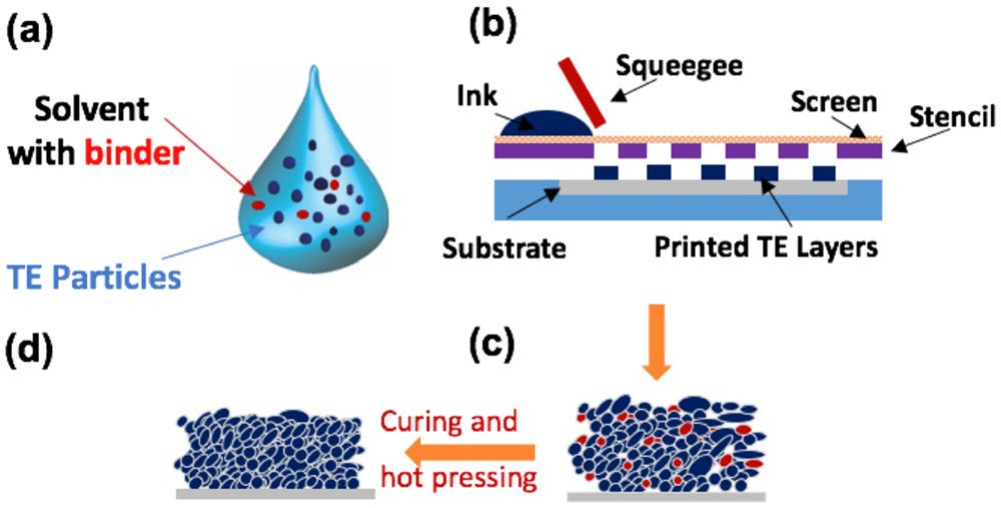
Schematic illustrations of (**a**) printable ink, (**b**) screen printing, (**c**) a screen-printed thermoelectric layer and (**d**) a hot-pressed layer after printing.

**Figure 2:**
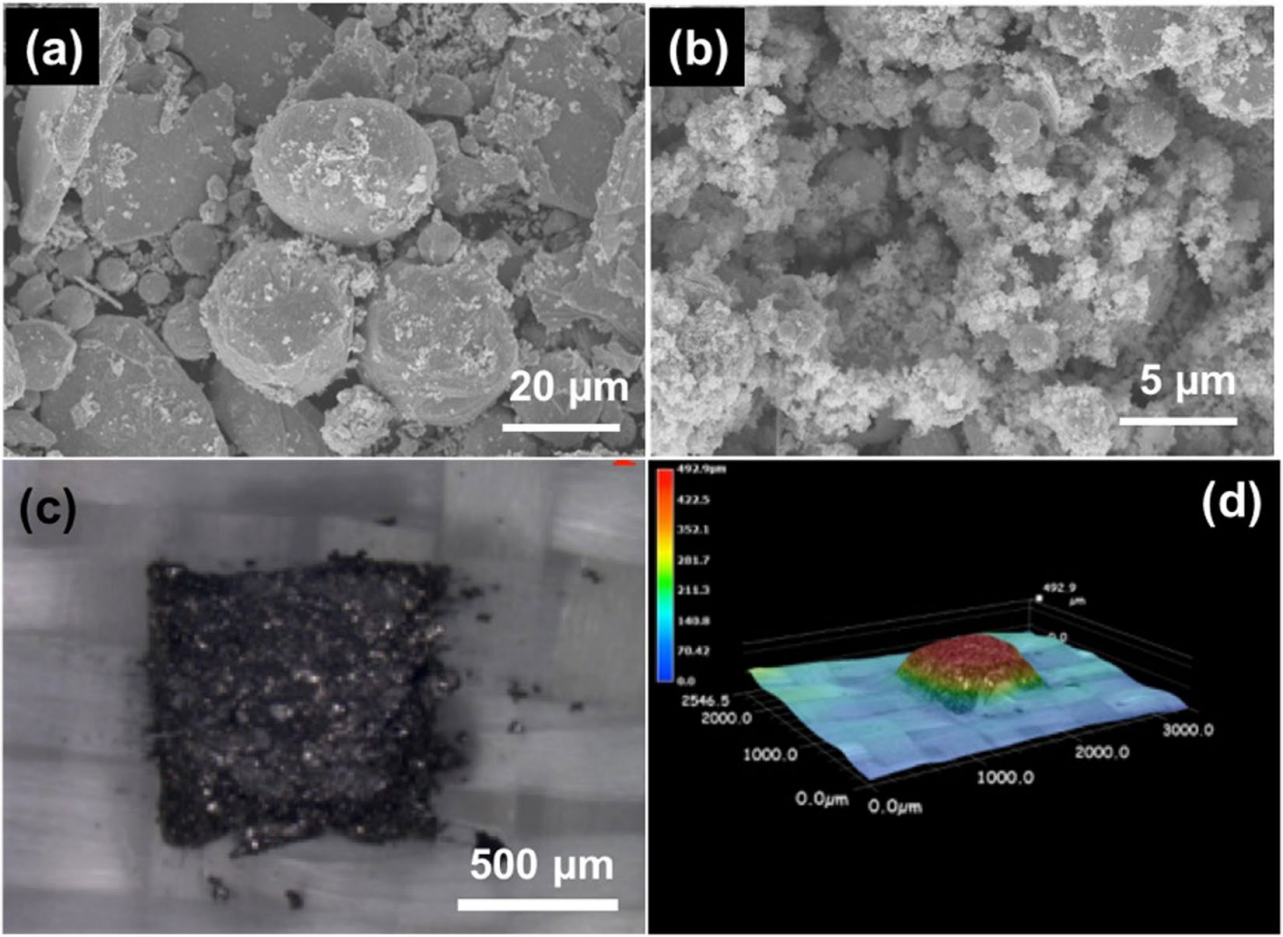
SEM images of particles with (**a**) large (>20 µm) and (**b**) small diameter (≪1 µm). Optical images of (**c**) printed thick thermoelectric ink for high aspect ratio TE pillars before the hot-pressing process, with 1 mm^2^ in area and 500 µm in thickness, on glass fiber fabric, and (**d**) Keyence 3D optical image of printed TE on the fabric.

Figure 1 shows the schematic of the screen printing process. We started with the fabrication of the TE particles using the spark erosion process, which can effectively break bulk TE materials into micro- and nano- particles, as reported in our prior publications^41, 42^. The starting bulk TE ingots have the compositions of Bi_0.5_Sb_1.5_Te_3_ for p-type (referred to as “BST” hereafter) and Bi_2_Te_2.7_Se_0.3_ for n-type (referred to as “BTS” hereafter), where ZT values at room temperature are 0.77 and 0.75, respectively (see Fig. S5). After spark erosion, the resultant particles display a bimodal size distribution consisting of large particles with >10 µm size and smaller particles with <1 µm size, as shown in Fig. 2. The particles used for printing were sieved through 45-µm meshes by taking the consideration of particle production yield and the uniformity of the printed layers.

We then prepared the printing slurries by mixing the active TE particles, organic binders, and solvent, as described in Methods. We selected Methocel as the binder because the cellulose ethers resulted in slurries with a viscosity of 3.3–3.5 Pa·s, which is within the optimal range to achieve uniform printing while minimizing the concentration of the binders (see Table S2 including the optical images of printed samples with various binders)^43^. Also, its low decomposition temperature makes it easier to be burnt out during sintering process (see Table S1). After printing and pre-heat treatment, TE powders and the binder were left on the fabric, as shown in Fig. 2(c). The as-printed TE layers had poor conductivity due to the loose contacts between the TE particles. Therefore, we used uniaxial hot pressing to densify the films, which reduced the thickness of the as-printed layers by approximately three times and greatly improved the conductivity. After hot pressing the printed layer on the fabric substrate, TE properties, including the Seebeck effect (*S*), electrical conductivity (*σ*), and thermal conductivity (*κ*), were evaluated at room temperature.

### Seebeck coefficient

**Figure 3:**
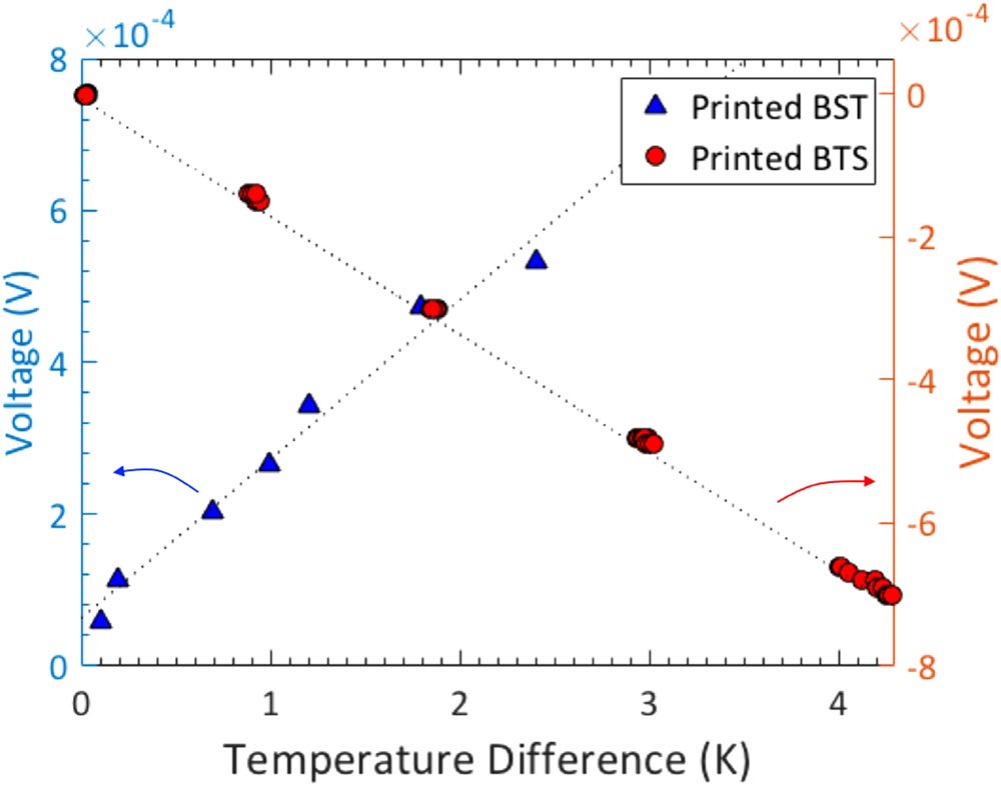
Plots of voltages as a function of temperature difference to obtain the Seebeck coefficient from the slops with p-type BST and n-type BTS.

Figure 3 shows the in-plane *S* coefficient measurement results, where the *S* is determined by the linear slope in the plot of thermovoltage as a function of temperature difference (e.g., *S* = *−dV/dT*). Our optimized slurries showed *S* of 209 µV/K and −165 µV/K in p-type and n-type materials, respectively after printing and pre-heating to remove solvents. The *S* value for the p-type printed BST is close to that of bulk BST (220 µV/K), whereas the value for the n-type printed BTS is considerably lower than the bulk value (−208 µV/K), presumably due to the composition change during the ink formulation or hot pressing process, as evidenced from the EDS analysis (see Figs S6 and S7). We also observed that the printed TE layers have similar *S* compared to that of TE powders, which were prepared by drop-casting the TE powders dispersed in organic solvents on a glass substrate followed by drying. This shows that the addition of the Methocel binder has minimal influence on *S*. This is because the Methocel is insulating and its amount is small (up to 0.6 wt.% of the inks), and *S* of the TE/Methocel mixture is dominantly determined by that of the TE materials.

### Electrical conductivity

**Figure 4:**
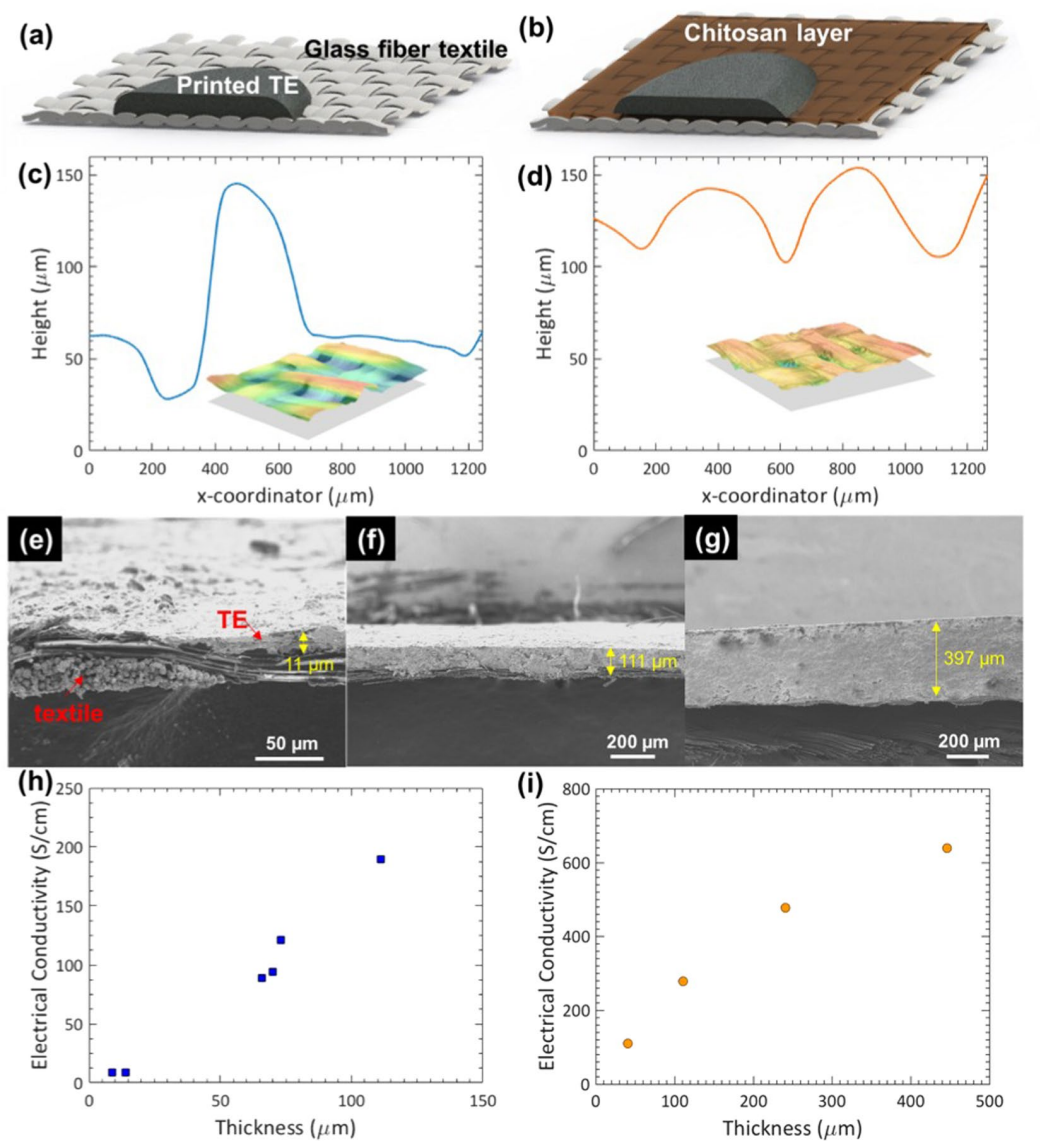
Schematics of printed thermoelectric layer on top of bare glass fiber fabric (**a**) and on Chitosan layer (**b**) used as a preliminary layer to smoothen the rough bare fabric. Optical surface profiles of bare glass fiber fabric (**c**) and printed Chitosan layer on top of glass fiber fabric (**d**) with their measured 3-dimensional surface images using a Keyence optical microscope (insets). SEM cross-sectional images of printed and hot-pressed samples on fabrics with thickness of 11 µm (**e**), 111 µm (**f**) and 395 µm (**g**). Plots of electrical conductivity as a function of thickness of the printed BST layers without (**h**) and with chitosan (**i**).

Due to the minimal influence of the binder on *S*, the main hurdle to achieve a high ZT in the printed films is the electrical conductivity σ and thermal conductivity *κ*. One of the challenges associated with the printing on fabric is the roughness and porosity of the substrate that lead to non-uniform printed layers, which would limit the electrical conductivity. Depending on the design of the weave and the material, fabrics have a certain degree of porosity and a large roughness over 100 µm (Fig. 4(a) and (c)), as determined using a Keyence optical surface profiler. This leads to the non-uniformity on the printed layers as some loose fibers can be embedded inside the printed layer as it cures. The infiltration of the TE material into the porous fabric also caused a large error in the determination of the layer thickness and consequently *σ*, especially for small layer thickness. As shown in Fig. 4(h), the *σ* of the printed BST layers on bare fabric was less than 200 S/cm, substantially lower than the bulk BST (*σ* ~ 1000 S/cm). Also, the *σ* increases with the thickness of the printed and hot-pressed TE layers: *σ* was improved from 8.5 to 190 S/cm by increasing the thickness from 9 to 111 μm (Fig. 4(e) and (f)), suggesting the detrimental effect of the substrate.

Our approach to mitigate the effect of the roughness and porosity of the fabric substrate is to print a non-conductive layer, referred to as the interface layer, to level the surface and block the pores in the substrate. We also increased the thickness of the TE layer to reduce the thickness ratio between the fabric and the TE layer and to minimize the adverse contribution of the fabrics. We adopted Chitosan as the interface layer, as schematically shown in Fig. 4(b). Chitosan is a well-known binder used in printing, e.g., for controlling the viscosity^44, 45^ and for treatment of fabric^46–48^. The presence of the Chitosan interface layer greatly reduced the surface roughness of the fabric, to within 50 µm, as shown in Fig. 4 (d). Consequently, the electrical conductivity was largely improved: at the same thickness of ~110 μm, employing the Chitosan layer increased the conductivity from 190 S/cm (Fig. 4(h)) to 278 S/cm (Fig. 4(i)). Thicker films printed on the Chitosan layers showed higher *σ*. We also carried out a similar study on n-type BTS by introducing the Chitosan layer and varying the printed TE layer thickness. As a result, the *σ* was improved up to 639 S/cm and 763 S/cm for p-type BST and n-type BTS on samples with Chitosan and thicker TE layers. Although the large thickness could sacrifice the flexibility of the TE layer itself, a device made of small printed TE pillars on the flexible fabric would still remain flexible.

### Thermal conductivity

**Figure 5:**
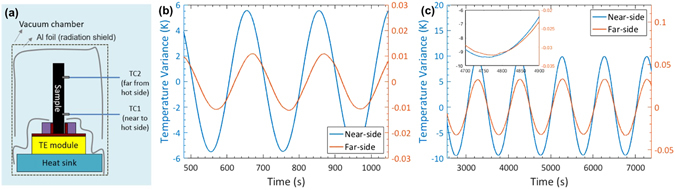
Angstrom method: (**a**) Schematic of the setup. Measured temperature wave of (**b**) p-type BST and (**c**) n-type BTS in the Angstrom setup.

Since *S* and *σ* were measured along the in-plane direction of the printed layers, it is imperative to also measure the thermal conductivity *σ* along the same orientation, which is often challenging. Here we employed the Angstrom method^49^ to measure the in-plane thermal conductivity (*κ*_||_) of the printed TE layers. In this method, one end of the sample was heated using a sinusoidal heat source, and the temperature waves were measured at two different locations of the sample, as shown in Fig. 5(a). We calibrated our setup with measurements on borosilicate and polyethylene (see Fig. S2 and Tables S3 and S4), which have well-known thermal conductivity values. The measured *κ*_||_ was found to be 1.29 and 0.77 W/m-K for the thickest (600–800 µm) p-type BST and n-type BTS layers (Table 1).

**Table 1:** Summary of thermoelectric properties of printed p-type BST and n-type BTS.

	P-type BiSbTe	N-type BiTeSe
*S* (µV/K)	209	−165
*σ* (S/cm)	639	763
*κ* (W/m-K)	1.29(*κ*_||_)	0.77(*κ*_||_)
	1.06(*κ*_⊥_)	0.83 (*κ*_⊥_)
*κ*_*L*_ (W/m-K)	0.97	0.37
ZT @ 300 K (using *κ*_||_)	0.65	0.81

To assess the anisotropy of the thermal conductivity, we also measured the cross-plane thermal conductivity (*κ*_⊥_) using the 3ω technique^50, 51^. By using the slope method (see details in the Supplementary Information), we determined the *κ*_⊥_ to be 1.06 and 0.83 W/m-K at 300 K, for p- and n-type TE layers, respectively. The in-plane and cross-plane thermal conductivity results suggest that the printed TE films were essentially isotropic after the uniaxial hot pressing process.

With the measured in-plane electrical conductivity and thermal conductivity, we can estimate the lattice thermal conductivity: *κ*_*L*_ = *κ* − *LσT*, where *L* is the Lorenz number, and is taken to be 1.67 × 10^−8^ *WΩ* *K* ^−2^ and 1.74 × 10^−8^ *WΩ* *K* ^−2^ for p-type BST and n-type BTS, respectively, where the *L* was corrected with *S* ^52^. The calculated *κ*_*L*_ is 0.97 and 0.37 W/m-K for the p-type and n-type layers, respectively. The *κ*_*L*_ for p-type BST is almost identical to that of the starting bulk material value (calculated to be 1.04 W/m-K by using the same *L* and the data shown in Fig. S5). However, the *κ*_*L*_ for n-type BST is significantly lower than the starting bulk value (1.23 W/m-K, see Fig. S5). This difference in *κ*_*L*_ in the p- and n-type printed layers is likely originated from the microstructures, as we shall discuss next.

### Investigation of microstructure

**Figure 6:**
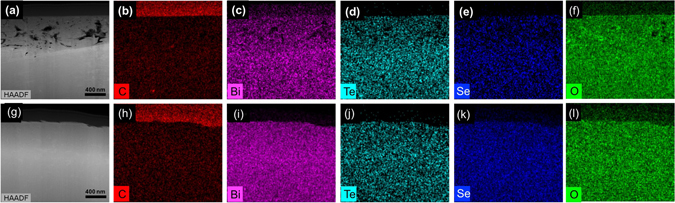
TEM EDS mapping images of hot pressed p-type (**a**–**f**) and n-type (**g**–**l**) with Methocel.

In order to investigate the relations between the measured thermoelectric properties and microstructures, high resolution-transmission electron microscopy (HR-TEM) was employed. The cross-sectional HR-TEM Energy-dispersive X-ray spectroscopy (EDS) element mapping images show the carbon element distributed in the p- and n-type TE layers in Fig. 6. In order to compare the amounts of carbon in the sintered samples, EDS quantifications were carried out. As shown in Table S7, the atomic percent of carbon in the samples with Methocel is similar to that in the samples without Methocel, revealing that the binders in the p-type and n-type were effectively removed without oxidizing the TE components and degrading the electrical conductivity. Therefore, the carbon elements observed with the binder in Fig. 6 were attributed to the hydrocarbon contamination in the surface of samples.

**Figure 7:**
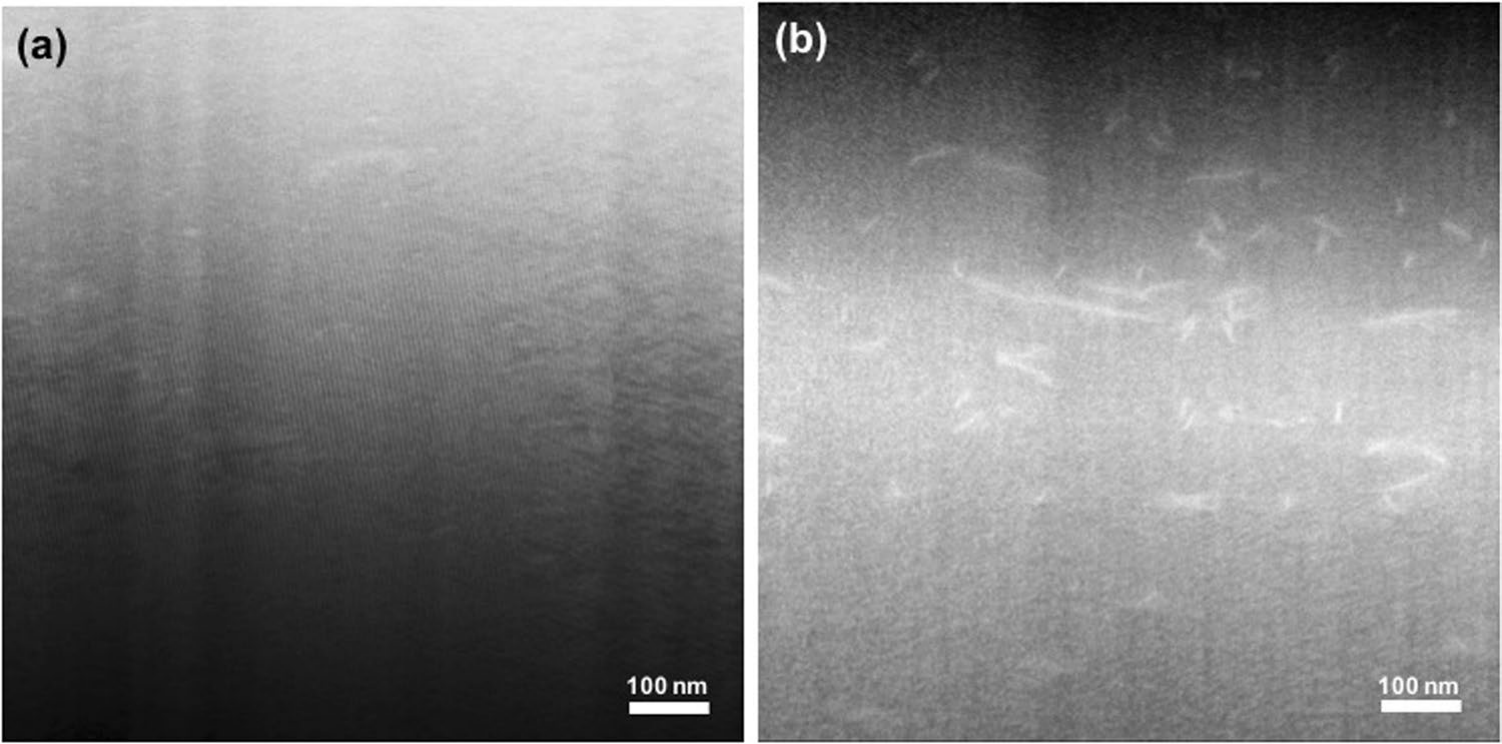
TEM HADDF images of hot pressed p-type (**a**) and n-type (**b**) with Methocel.

For the p-type printed sample, there is no clear nanoscale feature in the high angle annular dark field (HADDF) image in Fig. 7(a), similar to the case of hot-pressed p-type BST without Methocel. This may explain the similar *κ*_*L*_ between the p-type printed layers and bulk samples. However, for the n-type sample, the defects with the length scale of a few hundred nanometers were clearly observed in the printed sample with Methocel after hot pressing as shown in the HADDF image in Fig. 7(b). Considering that these defects were not found in the samples without Methocel (Fig. S4(b)), it seems that these thin and long defects were generated by the rearrangement of n-type TE particles while the binders were burning out in the sintering process, which could have facilitated the formation of the embedded defects. The nanoscale defects can effectively scatter the phonons in addition to the boundary scattering^53, 54^ due to the formation of spark-eroded particles, leading to lower lattice thermal conductivity in the printed n-type layers without significantly affecting charge transport. Thus, the thermal conductivity of the printed n-type samples is lowered compared to that of the bulk.

### ZT values

With the measured in-plane *S*, *σ*, and *κ*, we determined the ZT values for the printed TE layers to be 0.68 and 0.81 for the p-type and n-type printed layers, respectively, as summarized in Table 1. These values are among the highest for the printed TE samples. The high ZT values in our samples can be attributed to the following features of our processes: (1) we minimized the amount of the organic binder present in the printing slurries by using a high viscosity binder (Methocel). (2) The binder was also effectively burnt off during the curing and hot pressing process. (3) For the n-type printed sample, the nanoscale defects left behind after the binder burnt-off contributed to phonon scattering and thermal conductivity reduction, thereby leading to a high ZT value.

## Conclusions

We demonstrated printed TE layers with high ZTs for both p-type BST and n-type BTS (i.e., 0.65 and 0.81, respectively), which are close to the bulk values, on flexible glass fabrics. In addition to the high performance and the potential for flexible devices, introducing scale-up processes from preparing TE inks (e.g. spark erosion) to screen printing opens up the opportunities to adopt the TE generator for low-grade heat recovery in wearable devices and personalized thermo-regulation. In this study, we successfully addressed several critical issues commonly encountered in the printing of TE devices. First, we achieved high-quality printing using a small amount (0.45–0.60 wt.%) of Methocel, an insulating polymeric binder. Second, as the binder has a low decomposition temperature, we effectively burnt off the binders through sintering and hot pressing. The removal of the binders was evidenced from the microstructure analysis and the excellent electrical conductivity. Third, to ensure uniform printing, we also treated porous and rough woven fabrics by printing an interface layer using Chitosan, which enabled the printing of thick TE layers and improved the electrical conductivity. Moreover, we found additional benefit of binder decomposition: it created nanoscale defects that led to low lattice thermal conductivity for the n-type materials. The high-ZT TE layers achieved by printing on flexible fabrics reported in this work could lead to future development of low-cost flexible and wearable TE devices.

## Methods

### Spark erosion

We used the spark erosion method^41^ to fabricate TE particles. P-type and n-type bulk TE ingots were purchased from Thermonamic Inc. (China) with the nominal compositions of Bi_0.5_Sb_1.5_Te_3_ for p-type and Bi_2_Te_2.7_Se_0.3_ for n-type. The bulk ingots were shaped into two electrodes and smaller ‘charge’ pieces (~2 cm in diameter) that were immersed in a spark erosion cell filled with liquid nitrogen. The electrodes were connected to a charged capacitor that discharged high pulsed current and created sparks (micro-plasmas) between two ‘charge’ pieces with suitable distance. Large amount of particles could be collected at the bottom of the spark erosion cell.

### Screen printing and hot pressing

The TE particles were mixed with binder solvent which consists of methyl cellulose as the binder (METHOCEL HG 90, commercially available from DOW Wolff Cellulosics), in a mixture of ethanol 60 wt.% and water 40 wt.%. The concentration of the Methocel in the solvent was varied between 1.5 and 2.0 wt.%. The mass ratio between the TE particles and the solvent was 7:3, so Methocel is 0.45–0.6 wt.% in the final TE inks. This mass loading of the Methocel was found to offer suitable viscosity for screen printing. The slurry was thoroughly mixed using gentle ball milling for a day. Once ink is prepared, the ink can be applied to stainless steel stencil with a pre-design pattern laser cut with holes to deposit ink onto any substrate. The stainless stencil was design through computer aid design (CAD) software (AutoCad, from Autodesk). The designed pattern in AutoCAD was defined though a laser cutting service (Metal Etch Services, San Marocs, CA). The slurry was applied to the fiberglass fabric substrate (Fiberglass #00543 from Fibre Glast Development Corporation), through a stencil using an applicator. In some experiments, a Chitosan layer was printed first as the interface layer to smoothen the woven surface. The printed layer was cured at 250–300 °C for 30 mins to solidify the sample and also burn off the polymeric binders. Finally, the layer was hot pressed with a uniaxial pressure of 90 MPa at 450 °C for 5 min. The hot pressing was carried out in an Argon-filled glove box with oxygen concentration less than 80 ppm to prevent the oxidation of the TE layers.

### Electrical conductivity and Seebeck measurements

The TE layers after screen printing and hot pressing were approximately 1 cm^2^ and with thickness ranging from 10–700 µm. We used the van der Pauw method^55, 56^ to measure the in-plane electrical conductivity and a custom-made setup to measure the in-plane Seebeck coefficient. For the Seebeck measurement, the sample was placed across two thermoelectric blocks maintained at two different temperatures. Two T-type thermocouples were located near the two ends of the samples to measure the temperature. The temperature difference between the thermocouples was varied from 0 to 5 K, and the resultant Seebeck voltage was recorded with the two Cu probes in the thermocouples (Fig. 3). The Seebeck coefficient of the TE layers was corrected with the *S* of Cu (1.8 µV/K)^57^. This method was calibrated with bulk BST and BTS samples (Fig. S5) as well as a Ni foil sample (Fig. S2).

### Angstrom method

The Angstrom method^49^ involves the periodic heating at one end of the sample and the detection of the amplitude and phase of the resultant temperature wave at two different locations along the sample. In our setup, one end of the printed layer (approximately 1 cm long) was anchored to a Cu block, which was periodically heated using a thermoelectric module powered by a sinusoidal current. Two small thermocouples, one located near the heat source (near-side) and the other located further away (far side), were used two detect the temperature wave. The frequency range of the AC current was chosen such that the thermal penetration depth is as large as possible (to ensure a large and detectable temperature oscillation on the far end of the sample) but shorter than the distance between the two thermocouples. Under this condition, the thermal conductivity (*κ*) can be obtained as ref. 49:

1 $$\kappa =\frac{{L}^{2}}{2\cdot dt\cdot \,\mathrm{ln}(\frac{M}{N})}\rho {C}_{P}$$

where *L* = *x*_2_ − *x*_1_, *M* and *N* are the amplitudes of the temperature wave at *x*_1_ and *x*_2_, *dt* is the phase difference (in seconds) between *x*_1_ (near-side) and *x*_2_ (far-side). The measured thermal diffusivity was then converted to in-plane thermal conductivity (*κ*_||_ = *αρC*_*p*_) using the bulk specific heat values (*ρC*_*p*_ = 1.26 × 10^6^ and 1.22 × 10^6^ J/m^3^.K for Bi_0.5_Sb_1.5_Te_3_ and Bi_2_Te_2.7_Se_0.3_, respectively, based on data of Bi_2_Te_3_ and Sb_2_Te_3_ reported in ref. 58), where the contribution of the remained binders could be negligible owing to their little amount. Since the substrates are porous fiber glass having low thermal conductivity, we can assume that the measured sample *κ* is equal to that of the TE layers.

## Electronic supplementary material

Supporting Information
