# Supplementary material for: High-Performance Screen-Printed Thermoelectric Films on Fabrics
Source: Sci Rep. 2017 Aug 4;7:7317. doi: 10.1038/s41598-017-07654-2 (PMC5544726; doi:10.1038/s41598-017-07654-2)
Supplement: Supplementary file 1 — Supporting Information [file 41598_2017_7654_MOESM1_ESM.pdf]

Supplementary Materials for

High-Performance Screen-Printed Thermoelectric Films on Fabrics

Sunmi Shin[1], Rajan Kumar[2], Jong Wook Roh[3,*], Dong-Su Ko[3], Hyun-Sik Kim[3], Sang Il Kim[5], Lu Yin[2], Sarah M. Schlossberg[2], Shuang Cui[4], Jung-Min You[2],, Soonshin Kwon[4], Jianlin Zheng[4], Joseph Wang[1,2,$], and Renkun Chen[1,4, #]

[1]Materials Science and Engineering Program, University of California, San Diego, La Jolla, California, 92093, United States

[2]Department of NanoEngineering, University of California, San Diego, La Jolla, California, 92093, United States

[3] Samsung Advanced Institute of Technology, Samsung Electronics, 130 Samsung-ro, Suwon-si, Gyeonggi-do, 63272, Korea

[4]Department of Mechanical and Aerospace Engineering, University of California, San Diego, La Jolla, California, 92093, United States

[5]Department of Materials Science and Engineering, University of Seoul, Seoul, 02504, Korea

***Email: jw.roh@samsung.com**

$Email: josephwang@ucsd.edu

#Email: rkchen@ucsd.edu

1. Selection of binders

We would like to choose a binder that would lead to a suitable viscosity in the resultant ink with the minimal binder concentration. In addition, a low decomposition temperature is desirable, as it would mean effective binder removal during the sintering and hot pressing processes. According to

these criteria, we chose Methocel. Table. S1 shows the related properties of three binders we considered.

Table S1. Characteristics of potential binders for TE inks having low decomposition temperatures.

Name	Decomposition Temperature	Vehicle Composition
Methylcellulose (Methocel) 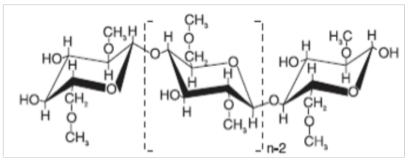	260-300 ^{o}C	1-2% in water/ethanol
Ethylcellulose (Ethocel) 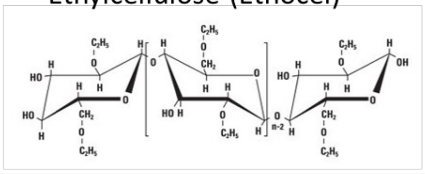	260-300 ^{o}C	5% in α-terpineol 10% in toluene
Polypropylene carbonate (Novomer) 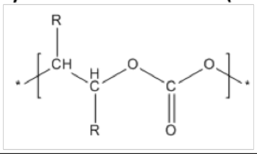	220 ^{o}C	15% in dibasic ester

2. Optimization of ink composition

We optimized the ink compositions, including the types of solvents and binders as well as their concentrations, in order to achieve suitable printability, which is assessed by inspecting the uniformity and morphology of the printed layers, as shown in Table S2.

The first binder used was polyvinylidene fluoride (PVDF), which has been commonly used in printable battery applications. We initially tried to have a concentration of 70% TE particles in a polar solvent, N-methyl-2-pyrrolidone (NMP), but the ink was almost entirely solid. In order to have a screen print ink, the concentration of solvent and binder needed to increase, which diminished the concentration of the TE particles to 30% with 50% of solvent and 20% binder. Table S2 shows the printability of the inks after screen printing and curing.

We then used a cellulose ether, trademarked as Methocel from DOW Wolff Cellulosics. This binder was used because of its low decomposition temperature, and the ability to solubilize in various solvents. We tried to replace the PVDF binder with Methocel at the desired lower

concentration but it required 20% of Methocel with TE particles remained at 30% and 50% solvent (NMP) to obtain the desired viscosity. We did notice an improvement of printing as shown in Table S2.

Table S2. The compositions of different inks using different solvents and binders, and images of their printability.

TE	Solvent	Binder	Image
70%	30% NMP	-	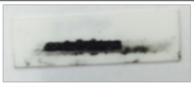
30%	50% NMP	20% PVDF	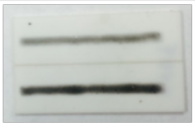
30%	50% NMP	20% METHOCEL™ HG 90	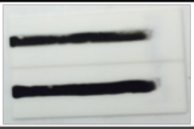
30%	50% NMP	20% PVDF	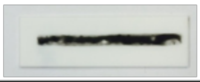
30%	50% NMP	20 METHOCEL™ HG 90	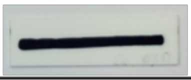
70%	25% NMP	2.5% METHOCEL™ HG 90	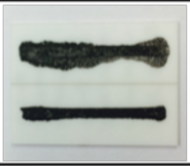
70%	25% EG	2.5% METHOCEL™ HG 90	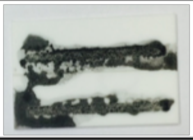
70%	25% NMP	5% METHOCEL™ HG 90	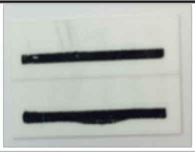
50%	50% (60% EtOH/40% H_2O)	2.5% METHOCEL™ HG 90	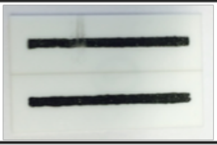
70%	30% (60% EtOH/40% H_2O)	2.5% METHOCEL™ HG 90	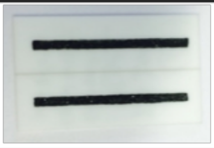
50%	50% (60% EtOH/40% H_2O)	1.5% METHOCEL™ HG 90	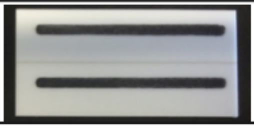

3. Calibration of the Seebeck measurement

We calibrated the Seebeck measurement by measuring the S of a 0.5 mm thick Ni foil. The Ni foil with 99.994% purity was purchased from Alfa Alsea. As shown in Fig. S1, the slope of the ΔV vs ΔT data is -20.6 μV/K. After subtracting the contribution of the Cu wires, the S of Ni is determined to be -18.8 μV/K, in excellent agreement with the literature value of -19 μV/K for Ni [1].

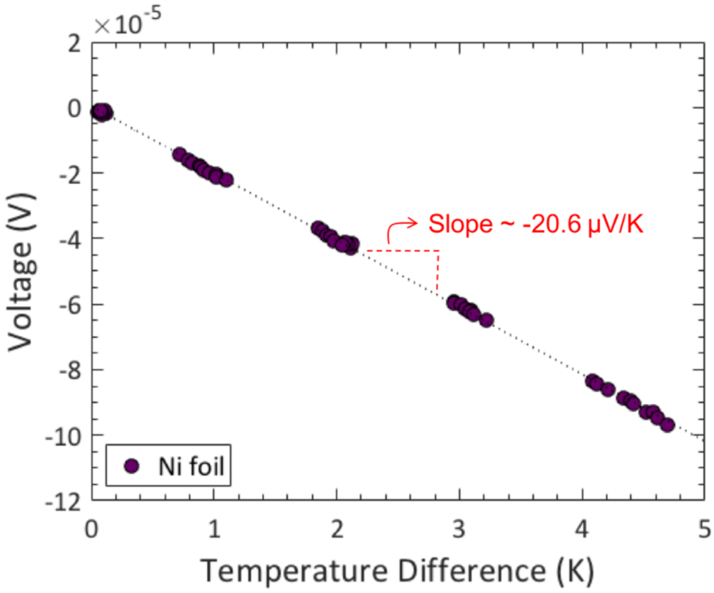

Figure S1. Open-circuit voltage as a function temperature difference for a Ni foil.

4. Angstrom method

We calibrated the Angstrom method using borosilicate and polyethylene samples, as shown in Table S3 and S4, respectively. To yield accurate measurement results, it is important to ensure a short thermal penetration depth (high heating frequency) as well as a short distance between the two thermocouples, compared to the length of the sample.

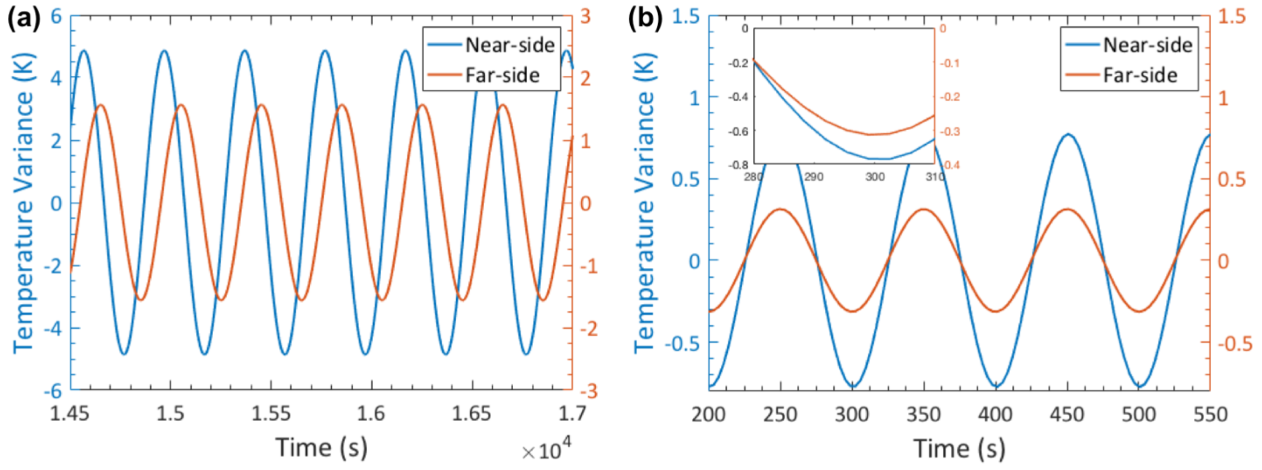

Figure S2. Temperature wave of borosilicate (a) and polyethylene (b).

Table S3. Summary of fitted values from Angstrom measurement on *borosilicate*

	@T_H	@T_C
Amplitude (K)	4.866	1.564
Ratio of Amplitude	3.111	
Frequency (Hz)	0.0025	0.0025
Phase (s)	68.067	151.700
Phase delay (s)	83.633	
Distance btw two thermocouples (m)	0.012	
Diffusivity (W/Jm^2)	7.586x10^{-7}	
Thermal conductivity (W/mK)	1.35	

Table S4. Summary of fitted values from Angstrom measurement on *polyethylene*

	@T_H	@T_C
Amplitude (K)	0.773	0.314
Ratio of Amplitude	2.461	
Frequency (Hz)	0.01	0.01
Phase (s)	25.840	125.111
Phase delay (s)	99.271	
Distance btw two thermocouples (m)	0.006	
Diffusivity (W/Jm^2)	2.01x10^{-6}	
Thermal conductivity (W/mK)	0.354	

Table S5. Summary of fitted values from Angstrom measurement on *p-type BST*.

	@T_H	@T_C
Amplitude (K)	5.485	2.504
Ratio of Amplitude	2.191	
Frequency (Hz)	0.005	0.005
Phase (s)	5.408	19.891
Phase delay (s)	14.483	
Distance btw two thermocouples (m)	0.005	
Diffusivity (W/Jm^2)	1.101x10^{-6}	
Thermal conductivity (W/mK)	1.29	

Table S6. Summary of fitted values from Angstrom measurement on *n-type BTS*.

	$@T_H$	$@T_C$
Amplitude (K)	9.696	7.614
Ratio of Amplitude	1.273	
Frequency (Hz)	0.001	0.001
Phase (s)	14.388	23.141
Phase delay (s)	8.754	
Distance btw two thermocouples (m)	0.00167	
Diffusivity (W/Jm^2)	6.59x10^{-7}	
Thermal conductivity (W/mK)	0.77	

5. 3ω method

We used the standard 3ω method to measure cross-plane thermal conductivity [2] (see Fig. S3(a)). The printed layers were first polished to a smooth finish as in Fig. S3(b) and then a thin (600 nm thick) parylene dielectric layer was deposited using the atomic layer deposition. Cr/Au 3ω heaters of 5 μm wide and 1mm long were fabricated through photolithography, sputter deposition, and liftoff (Figs. S3(c,d)). During the measurement, an AC current, $I(\omega)$, was applied onto the 3ω heater line which resulted in heat flux with a frequency of 2ω, accompanying with the generated thermal wave diffusing into the specimen. The penetration depth is determined by the thermal diffusivity of the specimen and the frequency of the AC current. Because the resistance of the heater depends almost linearly on the temperature, the resistance is also modulated at a frequency of 2ω. The voltage drop across the heater thus contains a third harmonic ($V_{3\omega}$) that depends on the AC temperature rise (T_2) of the heater, expressed as,

$$V_{3\omega} = \frac{I_0}{2}\frac{dR}{dT}T_{2\omega}\sin(3\omega t + \phi) \quad (1)$$

By extracting $T_{2\omega}$ from the measured $V_{3\omega}$ signal, we can obtain the thermal conductivity of the TE layers by the slope method [2],

$$k_s = -\frac{P}{2\pi L}\left(\frac{d(\Delta T)}{d(\ln(2\omega))}\right)^{-1} \quad (2)$$

where P is the heating power, L is the heater length, ΔT is the temperature rise. A length scale known as the thermal penetration depth can be defined from dimensional considerations,

$$L_P \equiv \left(\frac{\alpha}{2\omega} \right)^{1/2} \tag{3}$$

which physically represents the distance the thermal wave travels over the period of the heat oscillation. In order to satisfy the approximations made in the slope method, the fitting must be applied within a somewhat restricted frequency regime. To approximate an infinitely small line source experimentally, the penetration depth should be larger than the line width (which is finite in reality), and in order to justify the semi-infinite substrate approximation, the penetration depth should be shorter than the thickness of the substrate (which is finite in reality). The frequency range chosen for thermal conductivity a nalysis is from 1000 to 8000 Hz, which corresponds to the thermal penetration depth of 10 μm - 40 μm, smaller than the 3ω heater strip width (~5μm) used here.

Figures S3(e) and (f) show the measured 3ω voltage and the inferred temperature rise of the 3ω heaters on the p- and n- type samples, respectively. The responses at lower and higher frequency were related to thermal conductivity of the epoxy substrate and the TE samples, respectively. Based on the slope of the temperature rise in the higher frequency range, thermal conductivity of BST and BTS is 1.06 W/mK for p-type and 0.83 W/mK for n-type, respectively.

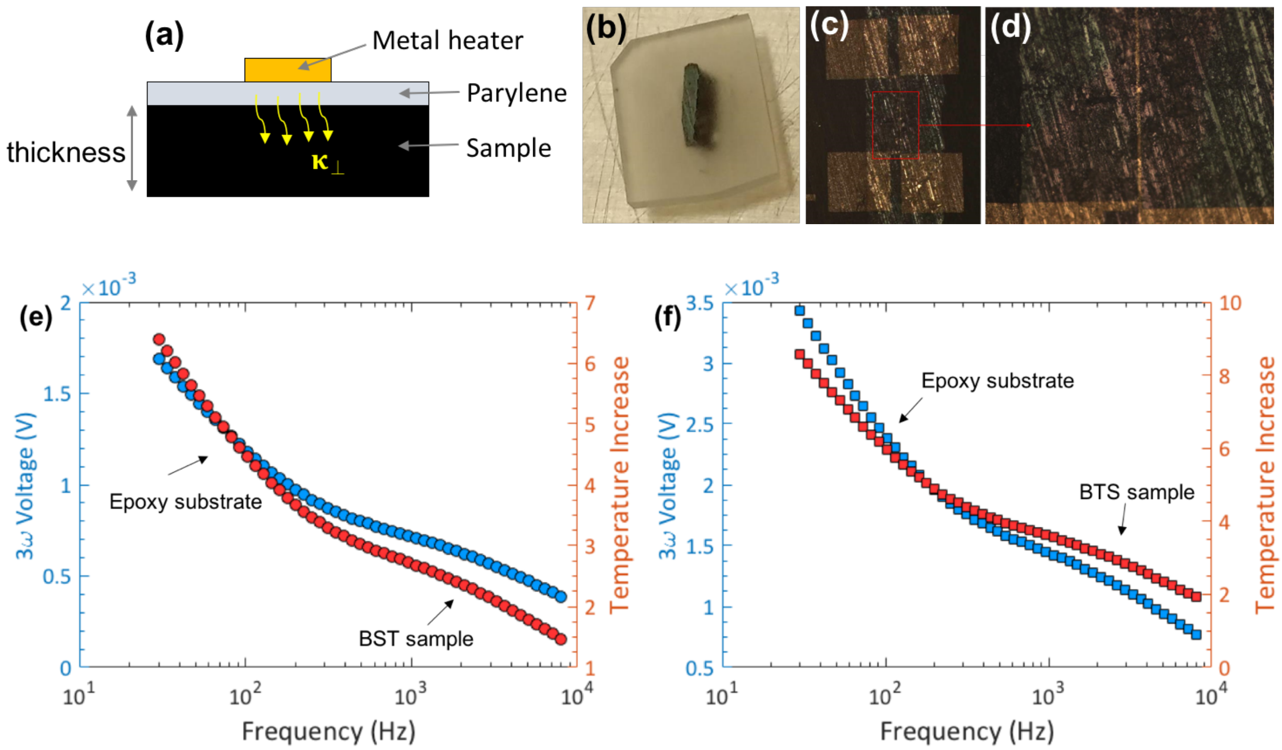

Figure S3. (a) Schematic of 3ω method for cross-plane thermal conductivity measurement. (b) Optical images of BST embedded in epoxy. (c,d) Optical images of the metal electrodes. Plots of measured voltages and temperatures of (e) p-type BST and (f) n-type BTS as a function of heating frequency using 3ω method.

6. Additional Microstructural Information

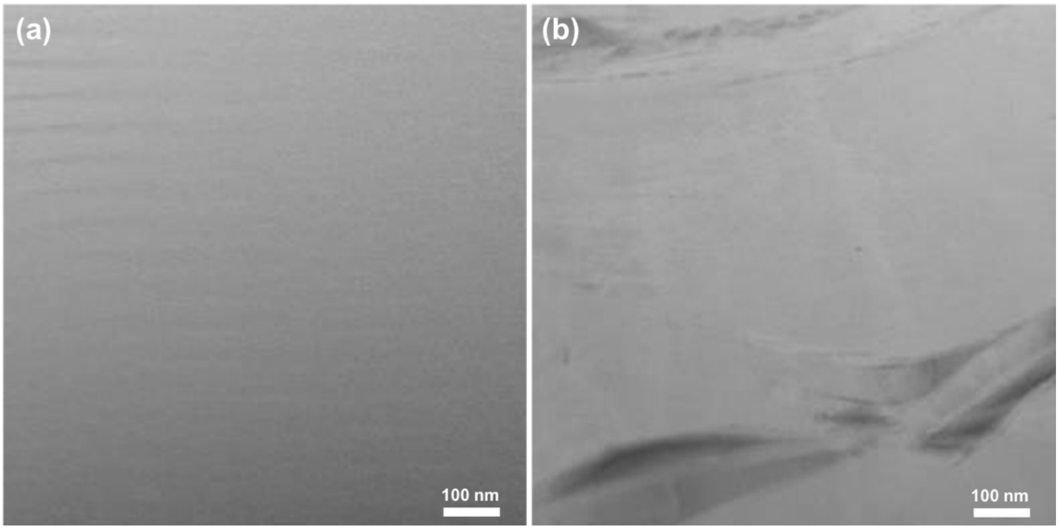

Figure S4. HAADF images of hot pressed p-type (a) and n-type (b) without Methocel

Table S7. TEM EDS quantification of hot pressed samples*

EDS quantification (atomic %)		Bi	Sb	Se	Te	C	O
p-type BST	without Methocel	14.44	28.57	-	51.43	2.11	3.48
	with Methocel	13.06	29.94	-	52.14	1.98	2.97
n-type BTS	without Methocel	43.56	-	3.85	46.50	3.73	1.29
	with Methocel	46.64	-	2.58	47.21	2.54	1.05

*Note: The TEM EDS was primarily used to compare the concentrations of C and O in samples with and without the Methocel binders. Since the TEM EDS analysis was only focused on very tiny localized areas and the samples may have non-uniform compositions, the compositions of Bi-Sb-Te in p-type and Bi-Te-Se in n-type from the TEM EDS analysis are different from those in the SEM analysis.

7. Thermoelectric Properties of Bulk Samples

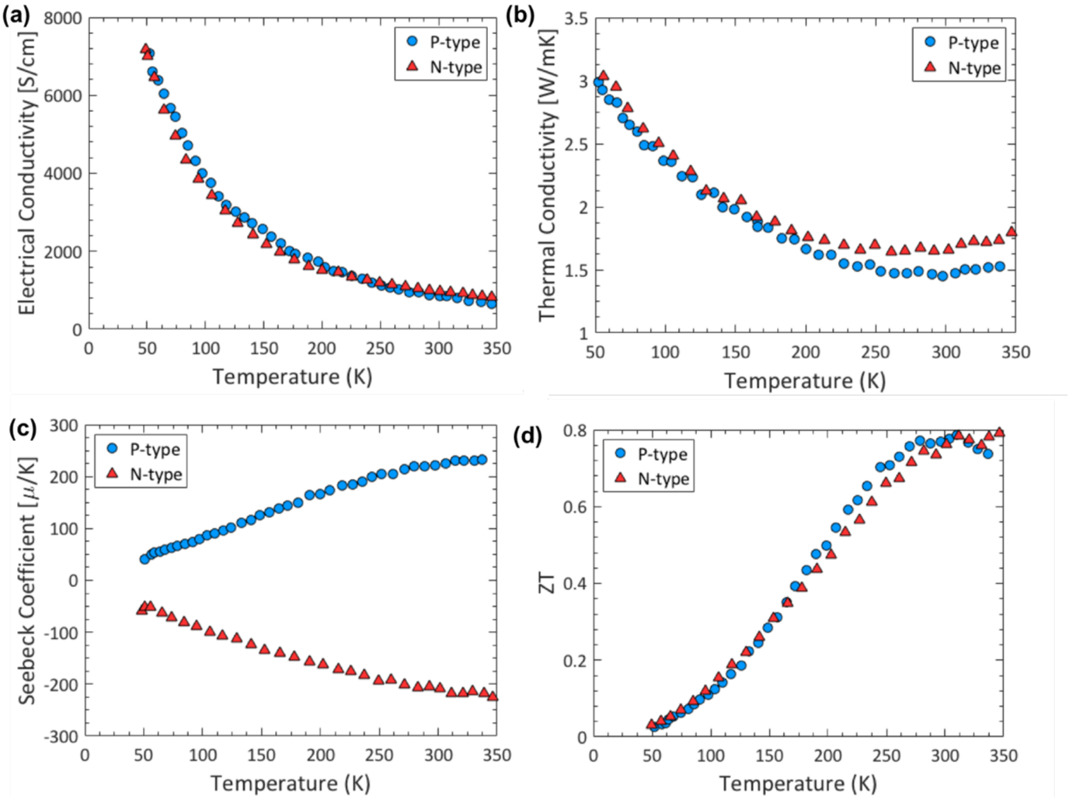

Figure S5. Plots of electrical conductivity (a), thermal conductivity (b), Seebeck coefficient (c) and ZT of bulk p-type BST and n-type BTS as a function of temperature.

8. Energy Dispersive X-ray Spectroscopy Analysis

Fig. S6 shows the compositions of the starting bulk p- type $Bi_{0.5}Sb_{1.5}Te_3$ and n-type $Bi_2Te_{2.7}Se_{0.3}$. To evaluate the composition variation, we carried out the EDS analysis on printed and hot-pressed samples, as shown in Fig. S7. We found that the concentration of Se was changed to $Bi_2Te_{2.85}Se_{0.15}$ in the n-type TE, while the composition remained the same for the p-type TE. Although the observed Se concentration change in the n-type sample is small, it cannot be negligible due to the fact that the optimized n-type solid solution ($Bi_2Te_{2.7}Se_{0.3}$) requires the low concentration of Se. It is known that the lower fraction of Bi_2Se_3 can reduce the energy gap, leading to a lower Seebeck coefficient.[3] Indeed, in our experiments, the change in the Se concentration led to the degraded Seebeck coefficient in n-type sample, from the bulk value of -208 μV/K to -165 μV/K in the hot-pressed sample.

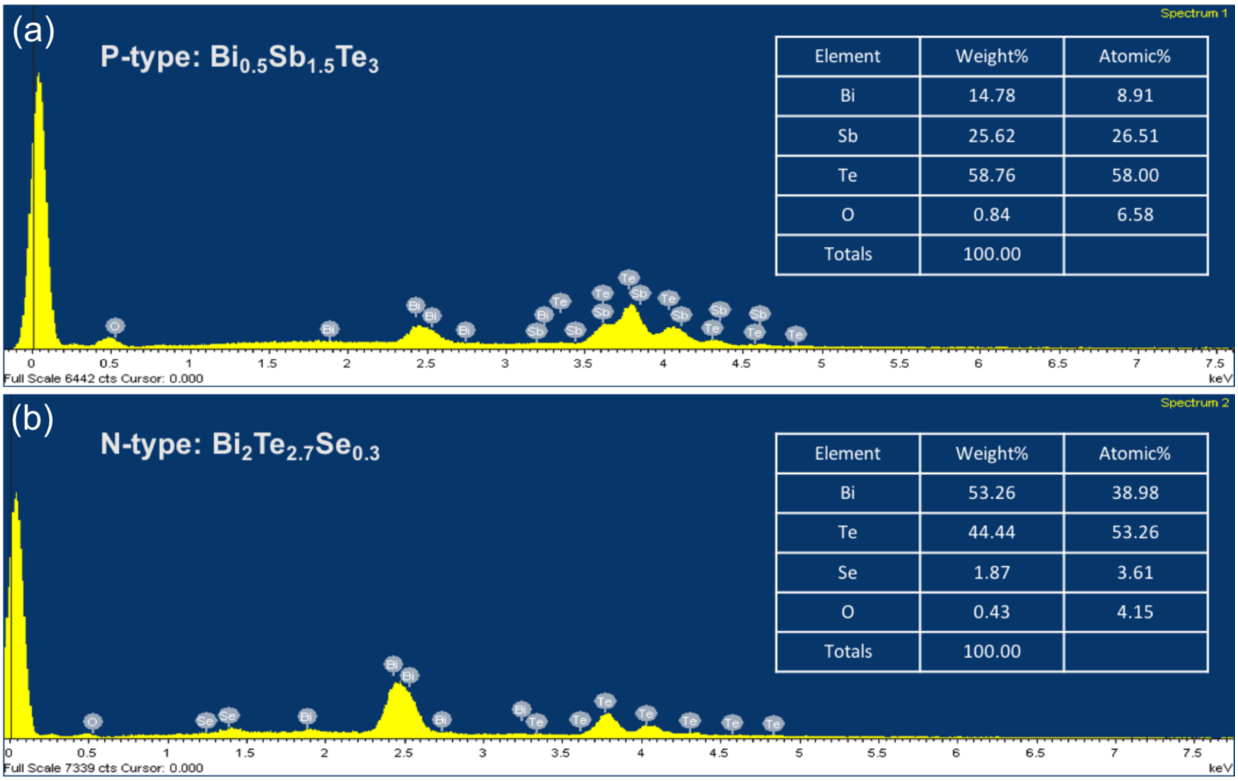

Element	Weight%	Atomic%
Bi	14.78	8.91
Sb	25.62	26.51
Te	58.76	58.00
O	0.84	6.58
Totals	100.00	

Element	Weight%	Atomic%
Bi	53.26	38.98
Te	44.44	53.26
Se	1.87	3.61
O	0.43	4.15
Totals	100.00	

Figure S6. Energy Dispersive X-ray Spectrum (EDS) of bulk p- (a) and n-type (b) samples.

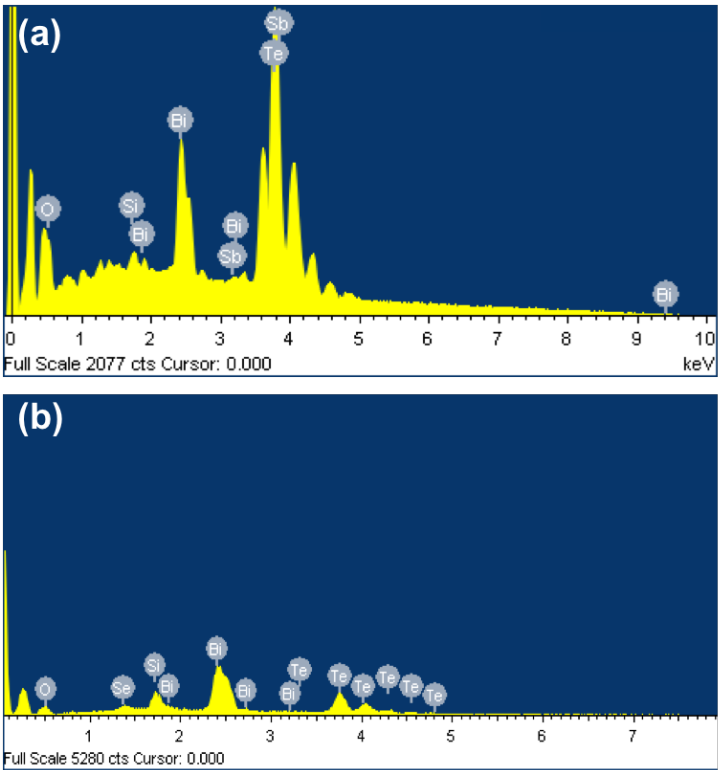

Element	Weight%	Atomic%
Bi	15.61	8.90
Sb	25.10	24.57
Te	57.36	53.58
Si	0.47	2.01
O	1.47	10.94
Totals	100.00	

Element	Weight%	Atomic%
Bi	51.25	32.01
Te	44.68	45.72
Se	1.58	2.60
Si	0.20	0.92
O	2.30	18.74
Totals	100.00	

Figure S7. EDS of hot-pressed p- (a) and n-type (b) TEs on glass fiber fabrics. The composition of

the p-type sample remained the same, while the composition of the n-type was changed to $Bi_2Te_{2.85}Se_{0.15}$.

References

1 Nakama, T., Burkov, A. T., Heinrich, A., Oyoshi, T. & Yagasaki, K. Experimental set-up for thermopower and resistivity measurements at 100-1300 K. *Xvii International Conference on Thermoelectrics, Proceedings Ict 98*, 266-269, doi:Doi 10.1109/Ict.1998.740369 (1998).

2 Cahill, D. G. Thermal-Conductivity Measurement from 30-K to 750-K - the 3-Omega Method. *Rev Sci Instrum* **61**, 802-808, doi:Doi 10.1063/1.1141498 (1990).

3 Greenaway, D. L. & Harbeke, G. Band Structure of Bismuth Telluride Bismuth Selenide and Their Respective Alloys. *J Phys Chem Solids* **26**, 1585-+, doi:Doi 10.1016/0022-3697(65)90092-2 (1965).
